# Citizenship in times of crisis: biosocial state–citizen relations during COVID-19 in Austria

**DOI:** 10.1057/s41292-023-00304-z

**Published:** 2023-05-22

**Authors:** Isabella M. Radhuber, Christian Haddad, Katharina Kieslich, Katharina T. Paul, Barbara Prainsack, Seliem El-Sayed, Lukas Schlogl, Wanda Spahl, Elias Weiss

**Affiliations:** 1 Department of Political Science, University of Vienna, Vienna, Austria; 2 Department of Science and Technology Studies, University of Vienna, Vienna, Austria; 3 Center for Global Health Policy, University of Sussex, Brighton, UK

**Keywords:** State–citizen relations, Biosocial citizenship, COVID-19, Pandemic policies, Austria, Qualitative interviews

## Abstract

Drawing upon 152 in-depth qualitative interviews with residents in Austria carried out in the first year of the pandemic, this article discusses how people’s experiences with COVID-19 policies reflect and reshape state–citizen relations. Coinciding with a significant government crisis, the first year of COVID-19 in Austria saw pandemic measures justified with reference to a biological, often medical understanding of health that framed disease prevention in terms of transmission reduction, often with reference to metrics such as hospitalisation rates, etc. Instead of using this biomedical frame, our interviewees, however, drew attention to biopsychosocial dimensions of the crisis and problematised the entanglements between economy and health. We call this the emergence of a *biosocial* notion of citizenship that is attentive to psychological, social and economic dimensions of health. Insights into the biosocial nature of pandemic citizenship open a window of opportunity for addressing long-standing social injustices.

## Introduction: citizenship in a public health crisis

The COVID-19 pandemic has posed novel challenges to societies and raised complex questions about how to navigate everyday life under pandemic circumstances. Drawing upon 152 in-depth qualitative interviews carried out in Austria at two time points (April and October 2020), this article examines people’s responses to COVID-19-related government policies. We find a shift in the understanding of health during the first year of the pandemic that problematised the narrow focus of government-driven pandemic policy: We saw a shift from a notion of ‘health’ rooted in relatively narrow, biomedical terms (aligned with the government’s focus on preserving health systems’ capacities) towards a broader biopsychosocial notion of health that is more sensitive to socioeconomic aspects of wellbeing. We further explore how people’s experiences with pandemic governance have reshaped our participants’ expectations of, as well as in relation to, the state in terms of what we call pandemic citizenship.

Social scientists have long explored how relations between states and citizens are affected by crises (e.g. Moon and Cho 2022; Boin et al. 2021; McCormick 2012; Kale-Lostuvli 2007; Boin 2004; Boin and ‘t Hart 2000, 2003; Hood and Rothstein 2001; Majone 2000; Jasanoff 1997; Bovens and ‘t Hart 1996; Epstein 1996; Wynne 1989). Empirical analyses have found that health—broadly understood—is an important arena for the formulation of expectations and claims vis-à-vis the state (Kieslich 2018). They have shown how citizens (re)negotiate relations to the state through their everyday practices and in their specific sociocultural contexts (Spackman 2018; Sharon 2015), and that relationships of trust between governments and citizens can increase the acceptance of public health measures (Jensen and Leibetseder 2021; Pfattheicher et al. 2020). In the sociology and anthropology of health and medicine, authors (Petryna 2002, 2004; Epstein 2008; Rose and Novas 2004; Wehling 2010) have explored ‘biological citizenship’ as newly emerging relationships between citizens and authorities. Highlighting the importance of biological perceptions for the political subjectification of individuals, they have put the focus on the role of somatic experiences and rationales for state–citizen relationships.

In this paper, we explore how people viewed pandemic policies and their own role in pandemic governance as citizens—meaning all people living in Austria, not only those holding residence permits and passports. Through people’s everyday experiences during the pandemic, we examine how people positioned themselves vis-a-vis the state in terms of what we call ‘biosocial pandemic citizenship’. The term pandemic citizenship is not new, but so far it has been used predominantly to theorise about what it means to be a citizen in a pandemic, especially within the confines of a nation state (Hollings 2020; Kruman and Marback 2022; Redmond and Xu 2022). By offering empirical insights from a large qualitative study, this article moves beyond the realms of citizenship theory towards an understanding of state–citizen relations that is grounded in how people experience, and make sense of, the challenges of the pandemic and the ensuing government measures. We draw on data from 152 in-depth qualitative interviews with a total of 80 people in Austria carried out in the first year of the pandemic (April–May and October–November 2020) to examine the perceptions of pandemic policies and what these tell us about developments in state–citizen relationships. By ‘pandemic policies’, we mean all policies and measures issued with the aim of containing the spread of the SARS-CoV-2 virus and of mitigating the effects of the pandemic. Broader than the notion of non-pharmaceutical interventions (NPIs), they include measures aimed at addressing the social and economic harms incurred by the crisis.

Our argument proceeds as follows: First, we situate our study in the literature on state–citizen relationships during times of crisis, where we also discuss the value and the limits of biological citizenship as a conceptual framework to describe the evolving state–citizen relations under the sign of the early waves of COVID-19 in Austria. We then describe the study’s methodological approach in answering the following research question: What do people’s perceptions of, and reactions to, policies tell us about changing/continuing state–citizen relationships? The findings section shows how people articulated their expectations and hopes towards the state in the first year of the pandemic, demonstrating a shifting understanding of health in this phase of the COVID-19 crisis. This shift is twofold: first, from a narrow biomedical to a broader biopsychosocial concept of health and second, from health as a concept that is posited against the ‘wellbeing’ of the economy towards health as a value that encompasses social and economic aspects of living well. The article ends with a discussion of these findings and reflections about the meanings and changes of citizenship during the COVID-19 pandemic.

## Understanding changing citizen–state relations during the COVID-19 pandemic

### The COVID-19 pandemic in Austria as a transboundary crisis

For the purpose of examining the ways in which the COVID-19 crisis influences state–citizen relationships, and due to its complexity and its scale, we understand the COVID-19 pandemic as a “transboundary” crisis (Boin 2019; see also Boin et al. 2021). As characterised by the failure of established mechanisms of crisis management, “transboundary crises pose a wide and deep challenge to the standing governance arrangements of democratic states […]. The state is left rudderless in a time when citizens look to their elected leaders and trusted institutions to navigate them through the storm” (Boin 2019, p. 96). Unfulfilled expectations regarding effective crisis management risk an erosion of government legitimacy that in turn decreases the capability of public institutions to solve the crisis.

Crises can lead to a seemingly paradoxical situation in which both the limits and expansion of governmental power moves into the focus of public attention (Villadsen 2021, p. 3; also see Bigo et al. 2021; Trnka 2021; Trnka et al. 2021; Abi-Rached 2021). They often expose pre-existing problems of governance (Paul 2012, for another example see Petryna 2002), pointing to the necessity of social and political change. At the same time, crises require that these existing institutions are functional, particularly in an area such as public health and safety measures (Béland 2005) that require comparatively strong state action. The tensions and contradictions inherent in this scenario are exacerbated when the states expand their powers in a state of emergency (Malandrino et al. 2022), while remaining unable to alleviate the pandemic at a national level. Such crisis moments, we propose, potentially challenge state-society relations and established understandings of citizenship.

Austria has a well-performing healthcare system: intensive care unit capacities are well-developed and statutory healthcare insurance covers over 99.9% of the population (OECD/European Observatory on Health Systems and Policies 2021). Only 12% of Austrian residents reported that they had to forgo medical care during the first 12 months of the pandemic compared to 21% in the European Union (OECD/European Observatory on Health Systems and Policies 2021, p. 3). The first national lockdown started on 16 March 2020. In line with similar measures taken in many other countries across the globe at that time, people were not allowed to leave their homes, with only four exceptions: Going to work, only in cases that preclude working from home; shopping for groceries and medicines; helping others who are in need; and physical or mental recreation outdoors, alone or with members of the same household (Republik Österreich 2020). While the government took an approach based on protecting health according to its predominantly biomedical understanding, compliance by citizens was high during this first lockdown (Mätzke 2021; Spahl et al. 2022). Measures to contain the virus were marked by governmental rhetoric prominently using virological and bio-epidemiological language such as references to virus loads and incidence levels, and were announced in regular press conferences, sometimes even daily (Czypionka and Reiss 2021).

The initial management of the virus outbreak was accompanied by the mechanisms of socioeconomic support not only to businesses via newly created financial aid instruments, but also to citizens through existing social security arrangements (such as *Kurzarbeit*, i.e. short-term work that helped reduce unemployment), leading to the easing of the measures in late April 2020 (Schmidt et al. 2020; Schmidt and Haindl 2021). While a wide majority of citizens perceived this initial pandemic management as successful (Krejca et al. 2022), the developments over the summer ended the initially successful approach.

A more fragmented approach to pandemic management marked the subsequent lockdowns and the policy measures to contain and mitigate the following ‘waves’ of infections (see Czypionka and Reiss 2021; Mätzke 2021). Austrian authorities declared a second and third strict lockdown between early November 2020 and early February 2021 (Pollak et al. 2021). The complex governance structure of the Austrian public health system, in which competencies are split between the federal and the state level, meant that pandemic measures across Austria varied: At times, some regions extended lockdowns while others were lifting them or introduced local measures, such as mask mandates for the unvaccinated (OECD/European Observatory on Health Systems and Policies 2021). Despite widespread availability, COVID-19 vaccination uptake stagnated in late summer 2021—followed by a slow increase from 74.6% in late September 2021 to 75.3% in late October 2021 of people who have received at least one vaccine shot (see Eberl et al. 2021; Paul et al. 2021, 2022).

It is important to add in this context that alongside the pandemic, a government crisis unfolded in Austria. Following the so-called Ibiza affair,^1^ a video recording of a high-ranking right wing politician offering favours to a presumed oligarch, the Austrian government had been dissolved in June 2019. After a brief interim government, a new government was inaugurated only 2 months before the first lockdown, in January 2020. As led by chancellor Sebastian Kurz, the centre-right Austrian People's Party (ÖVP) entered a coalition government with the Green Party. Since then, key members of the government—including the Chancellor, the Minister of Health, the Minister of Labour and Economy and the Minister of Interior^2^–became the faces of pandemic management in Austria.^3^ Commentators located a deep crisis of trust in the government and legitimacy by the end of 2021 (Zandonella 2021; Krejca et al. 2021). Reflecting earlier findings on risk regulation during crises (Hood and Rothstein 2001), declining legitimacy of the government and a subsequent diminishing capability of its institutions revealed the dual nature of the crisis.

The drawn-out nature of the pandemic *and* the government crisis combined to make Austria’s transboundary crisis particularly complex. Nevertheless, representative survey data for Austria show that agreement with the measures and the government was relatively high in the beginning, but decreased over time. For example, in late March 2020, more than 75% of the Austrian resident population said they trust the government, while only around 30% did so in October 2021 (Kalleitner and Partheymüller 2020; Kalleitner et al. 2021).^4^ This trend is similar to trends seen in other countries, where research on public attitudes towards various aspects of policy-making, for example scrutinising citizens’ perceptions of contact tracing applications (Lucivero et al. 2022), vaccination policy (Paul et al. 2022; Fiske et al. 2022) or face masks (Lupton et al. 2021; Pfattheicher et al. 2020; Schönweitz et al. 2022), shows a myriad of reasons for variations in compliance and non-compliance. These include various societal dynamics such as social movements shaping the countries’ responses to the COVID-19 pandemic in different regions of the world (Abers et al. 2021) and civil society organisations acting in alleviating the impacts of the pandemic (Kövér 2021; Dayson and Damm 2020). On a global scale, research has found that public perceptions of government responses to COVID-19 reflect the population’s trust in the government, people’s risk of exposure and also socioeconomic and democratic dynamics (Lazarus et al. 2020).

In Austria, people’s reasons for complying with the measures during the first lockdown were manifold. As noted, in the first months of the pandemic, there was a high degree of trust in the government (Laszweska et al. 2021; Kittel et al. 2021) and societal values were largely shared (Spahl et al. 2022). For example, trust in science, moral reasons, fear of infection and respect for legal authorities were decisive values that shaped how people acted upon the measures (Spahl et al. 2022). Strict lockdowns and punishing those who violate the rules, a rhetoric of fear and little emphasis on governmental communication including the lack of trust in the governed then proved to limit people’s satisfaction with governmental measures in Austria (Jensen and Leibetseder 2021).

This paper departs from a focus on what drives compliance with, and trust in, public health measures and instead explores how people *experience* government policies and how they *relate* these to broader societal concerns and expectations from the state as citizens. Contrary to the effects of earlier health crises such as the Chernobyl crisis described by Petryna (2002, 2004), we find that in the first year of the pandemic, citizens moved ever farther away from a somatic notion of health. People resisted a possible biologisation of citizenship and foregrounded biosocial notions of citizenship. Below, we unfold the conceptual repertoire that inspired our analysis, drawing on the literature on biological citizenship.

### Approaching state–citizen relationships through pandemic biological citizenship

Attending to the lived experience of people and the ways they make sense of current challenges is key when studying state–society relationships (Somers 1994). Conceptualisations of biological citizenship sensitise our understanding of how embodied (health) crisis experiences can lead to a (re-)articulation of citizenship. As developed by Adriana Petryna (2002, 2004), it refers to deeply personal experiences of crises and crisis governance that may prepare the ground for the forging of new identities within, and meanings of, state–citizen relations.

Petryna (2002, 2004) examined how citizens of the new post-Soviet state of Ukraine dealt with the fallout from the Chernobyl nuclear disaster—what we can understand as another veritable transboundary crisis (Wynne 1989). For these citizens affected by elevated levels of health-risk and biological harm, “knowledge about risk, how to deliver it, value it, became something of a political resource” (Petryna 2004, p. 254). Biological harm and related experiences of suffering were mobilised to claim eligibility to services and compensation and thus rudimentary forms of citizenship. Petryna coined the concept of biological citizenship (Petryna 2002, 2004) to capture the ways people saw themselves as biologically damaged subjects who were legitimately demanding redress from the new state, thereby significantly (re-)configuring notions of statehood and citizenship in the wake of the Chernobyl disaster (Petryna 2002, 2004).

By contrast, another strand of theorising biological citizenship foregrounds the reshaping of state–society relations. Building on Paul Rabinow’s (1996) concept of biosociality, Nikolas Rose and Carlos Novas (2004) pointed out how “a new space of hope and fear is being established around genetic and somatic individuality” (Rose and Novas 2004, p. 36; cf. Rapp et al. 2004; Gibbon and Novas 2008; Wehling 2010). Thereby, projects of biological citizenship can be led by the state, as well as emerge from the bottom up through citizens’ practices. As Beth Greenhough points out,

Biological citizenship can be state led, based in biopolitical beliefs about the state’s constituent population and interventions which focus on maintaining their health. It can also be citizen led, when individuals or communities draw on a shared biological identity to place claims on the state for support and recognition. (Greenhough 2014)

The state-led approach refers to the various “citizenship projects” that state authorities have pursued, that is to say, the ways state authorities have conceptualised and acted upon individuals as (good) citizens (Rose and Novas 2004, p. 439). In turn, such citizenship projects offer templates for individuals to identify and fashion themselves as citizens in particular ways—or, to reject and resist such ‘invocations’ of citizenship. Scholars have shown how people selectively appropriate certain parts of public narratives about healthy citizenship and dismiss others (Sharon 2015). Individuals and groups often aspire social and political inclusion and participation in the decision-making and governance of health technologies and other developments designed to affect them, in addition to access to medical services (e.g. inclusion of diverse populations in clinical research, Epstein 2008; or parents of children with genetic disorders, who seek more public attention through health activism, Fitzgerald 2008).

Importantly, Petryna’s original concept of biological citizenship has been developed against the backdrop of an institutionally demolished state in the wake of the nuclear fallout and the collapse of the Soviet Union, where people hardly had access to functional public services and health care. In that situation, a multiplicity of “citizenship projects” (Rose and Novas 2004) were evolving to shape the emerging relations between the state and its population, and radiation-related diseases have assumed the role of a symbolic “passport” to access state services. Frequently zeroing in on the AIDS pandemic as it plays out in low-income countries of the Global South, therapeutic citizenship elucidates how, e.g. “HIV status can be used to claim resources from the public or non-governmental organisation programmes” (Nguyen et al. 2007). In many cases, these claims to therapeutic citizenship are not limited to the state, but become re-territorialised (Rose and Novas 2004, p. 440) and involve “citizenship assemblages” that include local, national and transnational power brokers (Patterson 2016) that may include also corporate actors as targets of “pharmaceutical citizenship” claims (Ecks 2008). Discussing biological citizenship in the COVID-19 crisis in Austria, then, requires sociopolitical and conceptual contextualisation of our case study as situated within a wealthy European democracy with an advanced health care system and reliable welfare state (see in section 'The COVID-19 pandemic in Austria as a transboundary crisis').

For both low- and high-income contexts, biological citizenship has been subject to criticism for its implicit neoliberal undercurrent that individualises responsibility, undermines social welfare and solidarity and blurs the nexus between rights and responsibilities that had been enacted in national political and social citizenship projects. As Cooter (2008, p. 1725) notes, biological citizenship has often been “perceived as celebrating the market and individual consumers”. Further, these critical accounts identify the ways in which biological citizenship works towards “molecularising” not only the individual body but also the body politic, e.g. by foregrounding individual genetic risk factors that divert attention from social factors and broader living conditions that shape health and illness (Aarden 2018).

Hence biological citizenship is seen to encompass both individualising and collectivising tendencies (Rose and Novas 2004; Russel et al. 2016). Following Rose and Novas’ (2004) analysis, the individualising moment refers to the ways in which individuals begin to think of and act upon themselves through biological and somatic categories; in turn, these biological thought styles also enable forms of *biosociality* (Rabinow 1996; Gibbon and Novas 2008). The latter refers to collectives being held together by reference to a shared biological characteristic, e.g. a genetic trait, particular disposition for disease—or a shared vulnerability during a pandemic. Scholars have pointed to the way in which biological citizenship is negotiated in the context of political regulation (Ecks 2008) and highlighted the importance of communities when negotiating biological citizenship (Spackman 2018). With these conceptual sensibilities to individualisation and collectivisation, we now turn to the analysis of our data.

## Methods

This paper draws on interview data collected as part of the multinational comparative qualitative study “Solidarity in times of a pandemic (SolPan)—what do people do, and why?”. Ten European countries are part of the study (Austria, Belgium, France, Germany, Ireland, Italy, the Netherlands, Portugal, Switzerland and the United Kingdom). For the purposes of this article, we report only on the Austrian data which come from interviews with adults living in Austria independent of their formal citizenship. The study commenced in April 2020 shortly after the first lockdown measures were introduced in the majority of European countries. Its aim was to explore people’s views on and reactions to pandemic policies via in-depth, semi-structured interviews in several phases. The first phase (T1) of the interviews took place in April and early May 2020, the second phase (T2) was carried out in October and early November 2020. Our interview guides for both time points (SolPan consortium 2021b; Wagenaar et al. 2022) included open-ended questions to explore how the COVID-19 pandemic had affected the daily personal and work lives of our interview participants, or how they felt about the measures introduced by governments and public health authorities to manage the pandemic.

80 and 72 interviews were carried out in phases T1 and T2, respectively. All interviews were conducted online (using voice features only) or via telephone. Interviewees were recruited using a three-step process that began with convenience sampling (drawing on social media, personal and professional networks) followed by techniques of snowball and quota sampling (Bryman 2016). After each interview, the interviewer filled out a demographics table for the study in which key characteristics were listed. They included age, gender, family and employment status, highest educational degree, household income and rural/urban living situation to ensure demographic diversity of our sample (see Appendix). The interview sample also provides insights into the lived experiences of immigrant populations and across ethnic groups. However, this study refrained from systematically collecting information on immigrant status and ethnicity (Wagenaar et al. 2022). The study received ethics approval by the University of Vienna Ethics Committee (Reference Number: 00544).

The interviews were audio-recorded and transcribed verbatim. With regard to data analysis, we used the qualitative data analysis software Atlas.ti Cloud to manage and code our data. First, we filtered data in relation to our research question ('What do people’s perceptions about policies tell us about changing/continuing state–citizen relationships?'). Based on a coding scheme developed by a dedicated group within our project (SolPan Consortium 2021a), all interviews were tagged according to topics. This allowed us to select interview passages that spoke to interviewees’ perceptions of, and relationships with, the government and other public authorities during the pandemic. Second, all authors inductively analysed the filtered passages from our interviews, following an approach inspired by Constructivist Grounded Theory (Charmaz 2014). Regular meetings and discussions fostered our conceptual work, analysis and refinement. The excerpts of interviews quoted in this article were selected in a collaborative process and translated by the authors of the paper.

## Citizen–state relations during the COVID-19 pandemic

### A common position with government demarcating health from the economy

We now explore how respondents made sense of measures issued by the government at the beginning of the pandemic. We investigate what meanings participants assigned to these measures not only for their personal lives, but for society at large. First, we find that for our participants, the pandemic exposed the sheer scale of the need for political regulation, and the high degree to which these interventions affect personal, social, and economic life. As a young woman, living and working in an Austrian city stated:

And I actually find that relatively intriguing. In general, I find the discussion of this crisis very intriguing, because it affects so many areas of life, yes? Because everything has to be manoeuvred politically, because so many areas of life are affected. So in terms of economics, the private lives of all kinds of people have been affected. (T1 XX02^5^)

Mirroring this notion of collective affectedness, in the early phases of the pandemic (April–May 2020), our interviewees shared how difficult it was for them to reorient themselves. They mainly worried about people’s health risks when contracting COVID-19 and about protective measures. At the same time, however, many expressed trust in the government and felt that they were lucky to live in Austria rather than elsewhere as expressed by a woman living in the countryside:

Well, I have to say, I mean, for the authorities, for everyone, this is a total challenge and an almost unexpected new situation. And I simply trust in the crisis management of the Austrian government. And they simply have to find the right balance between protecting the population, protecting health and also keeping the economy running. (T1 XX05)

In this early phase of the pandemic, many of our respondents were willing to give government officials and public authorities the benefit of the doubt, considering the manifold uncertainties at the time. In addition to this recognition of pervasive uncertainty and corresponding governance challenges, we find that faith in the effectiveness of the Austrian health system was integral to trust in pandemic governance overall. People repeatedly expressed their belief that the Austrian health system was strong and well equipped in comparison with other European and non-European countries, feeling that

we are relatively well positioned with the health care system anyway in contrast to other countries [...] Yes, I think that in Italy they are not so well positioned in health care and somewhere in the Far East it is probably much worse; I think – or in India. (T1 XX09)

Moreover, these early phases of the pandemic were characterised by a widely shared understanding of health between government and citizens. While the Austrian government put forth a biomedical narrative on health that rested on metrics such as on hospitalisation rates, viral load, incidence, available ICU beds etc., our interviewees repeatedly reflected on health in such a rather narrow sense, too. This was expressed not only in an epidemiological register of the need to ‘save lives’, but also in an ethical register in which this specific notion of health was seen as an absolute value with an overriding priority over other societal values, such as economic performance:

Well, I am of the opinion that saving human lives should be valued more highly than economic success. (T1 XX02)

I mean, I think the most important thing is health, that they just make sure that not as many people get sick as they say, that there are no beds, or that the whole thing gets out of hand; and the other thing is just then, yes, for all the businesses and companies it’s just bad again, but they will also recover again, I guess. (T1 XX03)

It was quite common in the early phase of the pandemic to set the aim of protecting the (narrowly understood) health of citizens against the need of protecting or restoring the economy. This perception was shared among various people in different living situations (e.g. people living with and without children, people with high and low income) as exemplified in the following quotes:

And they just have to strike the right balance between protecting the public, protecting health, and also making sure that the economy keeps going. (T1 XX05)

I think in the crisis we will always see that we have two scales here, on the one hand with health policy interests and [on the other] with economic policy interests. And you can’t completely exclude both, because of course at the end of the day we’re heading for a huge recession [on one hand] and on the other hand, of course, in the end we have relatively few infected people, which is good. (T1 XX08)

Our data thus point to a shared understanding between citizens and the government in Austria during the first months of the pandemic, based on the acceptance of a notion of health understood in biomedical terms. While in these first months the survival of the health system (and the health of people in narrow terms) was prioritised, the economy (often understood in microeconomic terms envisioning firms, companies needing saving etc.) was relegated to second place. However, as we will see, this perception started to fade over the subsequent months.

### Diminishing trust in the government: experiences of individualisation and confronting fear

While public trust in the Austrian government remained high during April–May 2020 (Pollak et al. 2020), the first signs that this was about to change started to show. In this section, we show how our respondents perceived the use of fear in the government discourse during the pandemic. Interview participants also began to observe the uneven impact of the pandemic measures on the population as an issue that had been neglected in government discourse and policy. All in all, these developments contributed to a drastic decline in the population’s trust in the government already by October 2020. As explained in section 'The COVID-19 pandemic in Austria as a transboundary crisis', public support for pandemic measures decreased significantly from April 2020 to October 2021.^6^

While many of our respondents expressed fear about the medical consequences of contracting the virus, often with reference to vulnerable family members, for some respondents the government’s alleged use of fear in governmental discourses became an anchor for criticising pandemic management. Already in April 2020, some of our respondents had felt that the government was using fear to limit people’s freedoms during the healthcare crisis, and to get people to comply with pandemic measures—something several of our interviewees, though not all, were critical of. Our data show that particularly those with secure jobs and pensions worried about an increase in the government’s powers, and especially what this could mean for the protection of constitutional rights. Moreover, some respondents suggested that the use of fear of death and disease in the communication of government and public authorities served as a justification to restrict people’s freedoms—and at the same time to get them to see the solution of the crisis as their own individual responsibility. A woman who lives with her children in a big town reports that:

I get annoyed that, from the government’s side, […] from the beginning they instilled fear in me as a citizen and all the burden was put on me, and all the responsibility […]. I have to keep my distance, I am not allowed to infect myself, I am not allowed to infect others. (T1 XX03)

Some participants reported on what they experienced as fear-mongering by government officials and public authorities, which, in their view, was a way of the government to place the responsibility for getting through the pandemic on the shoulders of individuals. Recurrent references to the need for people to ‘take the situation seriously’ made them feel belittled and distrusted by government authorities. Another participant talked about the possibility of executive bodies introducing restrictive laws or ordinances while people were ‘kept at bay’, effectively paralysed by a sense of fear.

Our participants described an atmosphere of different kinds of fear, in which everyone is afraid of the consequences of the pandemic and government measures, but the perceptions about the nature of the threat are contentious. Many participants were afraid of the medical consequences of the virus and the government’s lack of more stringent measures, while others were critical of the use of fear by the government. In this atmosphere, participants reported a sense of being left alone with translating the government’s policies into their lives—moreover criticising the lack of clarity and the inconsistencies of government measures in the second interview phase, which took place just before the second national lockdown in Austria in November 2020. For example, they saw it as logically inconsistent that they had to wear a face mask in the hallways of public buildings (including schools) but were allowed to remove the mask once inside a room—or that nursing home residents were not allowed to leave their rooms or receive visitors while their caregivers were not regularly tested. These developments may have contributed to a loss of trust in the government, as people felt that insufficiently clear yet far-reaching and drastic measures were often imposed on them, with little regard for fairness.

Issues of justice became increasingly important to our respondents as the pandemic progressed, which also had bearing on understandings of health. In April–May 2020, only one interviewee explicitly reflected on the link between economic impact and health, using the example of people who could slip into poverty during the pandemic:

So for me, these are already things where I think to myself, this is actually madness that one accepts that hundreds of thousands of people are driven into poverty and poverty risk, so to speak, in the knowledge that everything also has health effects. (T1 XX09)

If I now have, so to speak, after this crisis, I don't know, 100,000 more poor children and children at risk of poverty, does that also cause damage to health - so, that’s what I mean by weighing it up, so to speak [...], yes. (T1 XX09)

As more interviewees had started to link socioeconomic conditions to health, they referred to concrete experiences of inequality during the pandemic, underlining the need to acknowledge the unique social challenges specific societal groups or individuals were facing. Our interviewees were also concerned over structural barriers limiting people’s possibilities to take on responsibility individually. For instance, a woman living in a big city expressed such concerns with respect to migrants:

I don't know how they [people from migrant backgrounds] are now in the multi-storey buildings, how they are when they are alone, when they are old […]. And actually, I would like to know how they are. If they have someone [...]. I don't know, [...], how they are, with their migration background, what information they have. Did they... I only know about ourselves... my friend, who runs a [doctor’s] practice, said that they didn't know what was going on at the beginning because they simply didn't have the translation [of the official policies issued in German] […]. (T1 XX04)

Many interviewees noted the population’s diverging socioeconomic conditions as well as linguistic barriers. Some interviewees, however, also blamed migrants for what they perceived to be non-compliance:

In my perception, the majority adheres to the guidelines [...]. There is also the fact that especially when you go for a walk in the evening, you tend to see young people, perhaps more young people with a migration background, who tend to stand together in small groups. Where I think to myself, [among them] there is probably not quite as much awareness of the potential danger that this poses. (T1 XX05)

A friend of mine is a director in an emergency room in a hospital. And he says, especially Turkish families, they don’t care that COVID exists. [...] They go shopping and all kinds of things because they don’t care. They’re just, it's a different culture. (T2 XX06)

Racist or discriminatory views, assigning blame for rising infection rates to specific (especially migrant) communities, were visible in some interviews. Other interviewees, however, were more nuanced in talking about the role of different societal groups as compliant or non-compliant actors in the pandemic. It was noted that access to information and also other factors that determined people’s ability to comply with rules were important barriers that did not get enough attention by the political decision makers. Hence, people’s possibilities to comply with measures and deal with day-to-day challenges shape the contestations of pandemic policies.

We conclude that our respondents’ dichotomous view on the relationship between health and the economy gradually started to weaken as people shifted their attention away from narrow economic policy questions such as saving companies and businesses towards broader socioeconomic issues and the entanglements between health and economy. They began to highlight the link between economic impact and health by turning their attention to instances of inequality and the multiple structural barriers to political representation or social protection it creates. The importance of the economy, and of state-sponsored socioeconomic support for vulnerable populations, gradually replaced the view that health was only a biomedical matter. Instead, interview respondents reflected upon fair economic conditions as a crucial dimension of good health, given the many negative socioeconomic and biopsychosocial consequences of lockdowns. Thus, in the second interview phase, we saw a more holistic understanding of pandemic governance, in which health and the economy are entangled with reflections about social inequalities.

### Shifting perceptions (of health): experiencing inequality in a social pandemic

In contrast to April–May 2020, in October, people were increasingly concerned about knock-on effects of pandemic measures and brought their concerns about treatment delays and mental health into discussions about their own and others’ health. A woman just over 60 living in an urban area described her views as follows:

We have these increased heart attack deaths. We have a lot less cancer treatments. We’ve totally cut back on beds in psychiatry. [...] People who need treatment for other illnesses, i.e. serious illnesses, are not accepted. That’s already the case again. With all the consequences for these people. (T2 XX03)

While few respondents mentioned the mental health impact of the pandemic response in April 2020, this became a prominent topic 6 months later. Examples include references to mental health impacts in nursing homes, mental health issues arising from unemployment and the consequences of school closures. As one interviewee puts it: “… you can't ignore that, and the government should also focus on that. So that you don't just look at the health aspects and the economic aspects, but also the mental aspects”. (T2 XX08)

In this way, notions of public and individual health grew beyond the initial biomedical definition that rested on the shared value of ‘saving lives’ threatened by the virus itself. Rather than merely moving on to concerns about the ‘collateral damage’ done by pandemic measures, our respondents often supported pandemic measures while, at the same time, articulating a need for a pandemic response that acknowledged also people’s mental health-related, social and economic needs. The latter was often phrased in quite nuanced terms as many of our respondents reflected on the diverging experiences of people grounded in their socioeconomic living conditions. Interestingly, respondents from a wide range of socioeconomic backgrounds shared that they were doing well overall during the lockdown periods—if only because they had a dog that they could walk, or a small balcony. However, our respondents also pointed out that people’s situations varied widely and expressed concerns, for example, about people in precarious employment during the lockdown. One young man from a middle-income background who lives and works in an urban area expressed this concern as follows:

Many people who generally don’t have that much money [and who] will suffer a bit more from the pandemic if they lose their jobs, and so on. Or people who have precarious employment, I believe that they will suffer a lot. And that in the long term, depending on how quickly the economy picks up again, I think that for people like that it can be really hard in the medium term that they now have to use up their savings and that they won’t be able to get new jobs so quickly. (T2 XX02)

Some respondents pointed to peoples’ restricted possibilities to deal with day-to-day challenges during the pandemic. They perceived an imbalance between the government’s justification of invasive and restrictive measures on the basis of fear on the one hand, and political leaders’ unwillingness to adequately consider the social effects of pandemic policies on the other. Increased vulnerability for some groups and precarious situations limited not only compliance, but also peoples’ ability to adapt to day-to-day challenges. A woman living in a big city reflected on how pandemic measures affected parents—especially women—and their children:

I don’t know if the decision about the closures was […] the right one. But because the decision was made to close the parks, many more accompanying measures should have been taken. Women had so much additional suffering because of the additional burdens, and I have the impression that no good concept was worked out during the summer vacations. [...] Fassmann [then Minister of Education of Austria] and Co. didn’t care at all, that's my impression, how the children and also the parents and especially the mothers with the children were doing. (T2 XX04)

When several interviewees reflected on how difficult the intersection of care, family, and home-based work is for parents, women and children, they pointed out that the social consequences entail health *and* economic consequences. One woman living in an urban area with children shared that “…in the end it didn't matter at all, that's my impression, how the children and also the parents and especially the mothers with the children fared. […] I can only tell you what I perceived of the impact and of the 100% failures in the policy” (T2 XX04). She recounts that the government’s provision of laptops for students was an insufficient measure to ensure that students

have laptops, their own room or desk or whatever; and the many children who are already very disadvantaged in our education system and that by these Corona closures are still much more [...] that has quite certainly social, health and ultimately also economic consequential damages. (T2 XX04)

Accordingly, our respondents also saw the need for more social, psychological *and* economic support afforded to particularly vulnerable groups, such as the elderly, people with existing health conditions, but also children and their parents, women, students, people working in culture and the arts, and the precariously employed. Some of these worries related to the elderly growing lonely due to isolation requirements and contact restrictions, while people also feared that children would fall behind irreversibly in their learning and social development.

Our respondents’ view of ‘health versus the economy’ thus shifted towards an understanding of (socio)economic conditions as part of a broader understanding of health. Our interviewees also articulated an understanding of the economy that was much more centred on the socioeconomic conditions of individuals and groups in different living and working conditions, rather than predominantly on rescuing firms and companies. As a self-employed man with a relatively high-income living in a rural area with children put it:

Yes, I think so, on the one hand I don’t want the economy to continue the way it is going, into a growth etc. and the environment is broken. On the other hand, I am also aware, I do believe, that through these rescue packages, which exist throughout Europe in the various countries, companies are currently still being propped up, but there will probably also be a chain reaction of insolvencies and unemployment, and what consequences does that have for public health, what consequences does that have for people. What consequences does that have for children, for tensions in families? We don’t want that either. (T2 XX03)

As the pandemic continued, the way our respondents’ made sense of the notion of health and the economic challenges shifted from a narrower, biomedical notion of health to a broader biopsychosocial notion of health. In the second interview phase, health was increasingly seen as a multidimensional concept of wellbeing that encompasses medical, social, psychological and socioeconomic aspects. This articulation of health can be interpreted as a small, but consequential, shift in underlying notions of citizenship during this health crisis. The sustained experience of living in a pandemic, and the increasingly incoherent policy measures, exposed a chasm between people’s everyday experiences and the problems that government measures attempted to target. The multi-faceted reflection about the relationship between health, the economy and inequalities was not only accompanied by criticisms of government policies, but also by biosocial demands and citizen expectations.

## Discussion: citizenship in the context of the COVID-19 pandemic

In the context of the COVID-19 pandemic, communication from public authorities and decision makers in Austria has strongly revolved around a biomedical understanding of health, couched in medical terms such as proteins, viruses, antibodies and metrics. References to this technical understanding of health became the justification for a wide range of state-imposed measures, ranging from school closures to mask mandates to travel restrictions and vaccine prioritisation policies. This did not, however, lead to a biologisation of citizenship in the sense that the biological realm became an arena for the articulation of demands from, and responsibilities towards, the state. Instead, most of our interviewees drew attention to a wider range of collective issues—including medical, social, psychological and socioeconomic problems—that the government was supposed to solve, but allegedly decided to ignore (also see Marmot 2020; Marmot et al. 2020). They referred to the unequal social effects of pandemic measures as well as people’s divergent abilities to comply with them and deal with day-to-day challenges (also see Hassan et al. 2021; for the multiple burdens on women see, for example, Wöhl and Lichtenberger 2021). Our analysis points to a need for critical reflection on the dominance of unduly narrow conceptualisations of health as a source of moral and political justification for policies—and as one of the factors contributing to the declining public support for pandemic management and pandemic measures. Metzl and Kirkland (2010) provide an avenue for such reflection by highlighting the moral politics and power relations that are at work when ‘health’ is invoked as a seemingly neutral and unproblematic policy objective. We now turn to discussing newly emerging citizen–state relations that are rooted in the experience that health is more than a medical matter.^7^

### Newly emerging citizen–state relations during COVID-19 in Austria

As noted, in the first year of the COVID-19 pandemic, a technical and biological understanding of health was the most common justification for a wide array of measures ranging from the closure of schools as well as shops, restaurants and other businesses, to rules about physical distance, face-mask wearing and restrictions on the individual freedom of movement. Our participants began to question this understanding over the course of the first few months of the pandemic. In the very early phases of the pandemic, frustration was matched by a sense of gratitude among many of our respondents that the state was doing a relatively good job of managing the pandemic and keeping infections at bay. Yet this (relative) balance between frustration and gratitude gave way to increasing levels of frustration and resistance in the later months.

Our interview partners felt that responsibilities for pandemic containment were individualised and that they were left alone in dealing with the challenge of how to interpret pandemic measures, and how to translate and implement them in their lives. Some respondents described their frustration as what they saw as fear-mongering by the government to justify restrictive measures in the name of public health. This resonates with normative stances that criticise governments’ use of fear during the pandemic to build the basis for the illegitimate suspense of democratic principles (for example Dodsworth 2021). Within political theory, fear as a strategic instrument of politics has traditionally been associated with autocratic rule and considered to be irrational, as exemplified in the thinking of Martha Nussbaum and Hannah Arendt (Degerman et al. 2020). But fear also plays a role in democracies, albeit in a different way. Judith Shklar, for example, argued that the principle of the liberal state is to protect its citizens from legitimate fear about their biological and economic livelihood, so that they can live in freedom (Shklar 1989). In the pandemic, scholars have conceptualised fear as a useful and rational emotion (e.g. Degerman et al. 2020). Johnson et al. (2021, p. 320) argue that for the “effective management of crises, fear must be sustained alongside the promotion of civic virtues, such as solidarity, responsibility and duty”.

Examining the argument empirically that fear can have an instrumental function in times of crises, we found that citizens related to anxiety in multiple ways: most openly articulating their worries regarding the medical consequences of the virus and some criticising the use of fear by the government (though not all, as some also referred to a legitimate use of fear during the pandemic). Among those who criticised governmental fear-mongering, concerns regarding potential negative effects for democratic institutions were articulated, for example if fear is used as a pathway to expanding the powers of the executive. Much like the nuanced academic debate about the role of fear in political systems and in times of crises, our interviewees’ references to fear were manifold: from anxieties about the possible medical effects of the virus to concerns about the expansion of executive powers; and from criticising the governmental use of fear to finding it legitimate in a pandemic state.

Our data suggest that the focus of people’s expectation vis-à-vis the state shifted from the narrow health arena to a broader health–psychosocial–economic nexus towards the end of 2020. This shift was accompanied by an increase in people who did not comply with measures they did not understand or that they deemed unfair. This increase in non-compliance, and our respondents’ reflections on social inequalities, weakened the trust (Plescia et al. 2020) that seemed to be at the core of state–citizen relations in the beginning of the pandemic and gave way to the emergence of bio*social* citizen claims vis-à-vis the state.

### Towards biosocial citizenship in times of (health) crises

In our results, we can observe a double shift in our interviewees’ understandings of health, which opens a field for articulating a new kind of biosocial pandemic citizenship. While scholars have worked on biosocial activism and worlds and the ways in which these are related to citizenship (Valle 2015), the term biosocial citizenship has not yet been used to the best of our knowledge. On the one hand, we see a significant shift in people’s perceptions from April–May to October 2020, with people realising the consequential effects of pandemic measures, such as delayed treatment of other illnesses and increased psychosocial burden. On the other hand, we see a shift in perceptions of the economy relative to health; whereas in April–May 2020, our respondents spoke of the economy versus health and often understood the former in terms of companies and businesses that needed to be rescued, this changed in October 2020. Drawing on concrete experiences of inequality during the pandemic, our interviewees emphasised the socioeconomic conditions that affect people’s ability to cope and protect their lives and livelihoods, underscoring the interconnectedness of health and economic concerns.

Many of our respondents noted that these inequalities had existed even in pre-pandemic times, but observed that they were exacerbated through the pandemic. This is congruent with studies showing that different factors and dynamics of disadvantage and deprivation mutually enhance each other (e.g. Bambra et al. 2020; Horton 2020). Fiske et al. (2022b) show how health- and wealth-related inequalities intersect and produce a ‘second pandemic’ of inequalities, while Desai et al. (2020) explore how financial redistribution in social policy shapes state–society relations during the pandemic. In our study, we observed an acute awareness of peoples’ diverging positionalities and challenges during the pandemic leading up to social inequalities. Pandemic measures were seen as creating new inequalities around health in a broad sense articulated around people’s socioeconomic status, migration status, gender and others. People’s social positionality was something that they wanted the government to consider more prominently. Instead, however, they observed how the responsibility was placed onto individuals in spite of their differing social and economic positions and how massive undifferentiated support was provided, for example, to companies.

Our respondents highlighted the medical, social, psychological and socioeconomic circumstances that make the pandemic so difficult for people to bear—and for some much more so than for others. Taken together, these aspects can also be seen as prerequisites for good public health in times of crisis such as the COVID-19 pandemic. This distinction, and the debate about it, is not new in the academic community, not least because of the ongoing reflections about the usefulness of the WHO’s 1948 definition of health. To recall, according to the WHO: “Health is a state of complete physical, mental and social wellbeing and not merely the absence of disease and infirmity” (WHO 2022). Our research shows how citizens activate this understanding in extended health crises such as the COVID-19 pandemic, foregrounding a *biosocial* pandemic citizenship in times of crises.

## Conclusion

In this article, we have shown how citizens’ reactions were consistent with specific features of the COVID-19 crisis in Austria, such as its temporal evolution and concurrent political unrest. At the beginning of the pandemic, people’s trust in government was very high (a phenomenon that is known as the ‘rally-round-the-flag’ effect during crises; e.g. Mueller 1970; Erhard et al. 2021). A few months into the pandemic, as it became clear that the government would not be able to live up to people’s expectations, this changed. While the spread of the health emergency required institutions to respond fast and coherently, citizens’ trust in precisely these institutions was eroding.

Just as our interviewees began to criticise pandemic measures as not well communicated, inconsistent and lacking regard for fairness, government operations were interrupted by another incident. Rooted in corruption scandals prior to the pandemic, a government crisis blew up during the COVID-19 crisis in Austria. In this sense, deeper challenges within crisis situations became painfully visible during the pandemic in Austria, affecting the relations between citizens and the state in the country.

In the midst of Austria’s COVID-19 crisis, the perceptions, experiences and conclusions described by respondents in our study point to a questioning of the established relationship between the state and its citizens. In a context in which a medico-biological understanding of ‘health’ had become an ubiquitous reference point for government policies, participants in our study did not adopt a biomedical frame for the reformulation of their expectations from, and responsibilities towards the state. Instead, they called attention to the medical, social, psychological and socioeconomic dimension of the COVID-19 health crisis in Austria.

Contrary to the expert framing of the pandemic phase in medico-biological terms (Kraemer 2022), at least in its early stages, citizens soon came to understand the pandemic as *biosocial*, rather than merely biological. In this paper, we were able to identify a shift in our respondents’ perceptions from April–May to October 2020 from a narrow to a broader understanding of health, with this shift occurring in two steps. First, a biomedical understanding of health was complicated by observations about biopsychosocial components of wellbeing as people observed the psychosocial knock-on effects in relation to the pandemic and pandemic measures. Second, seeing other people suffer drew the attention of people to social and economic circumstances and raised concerns about the pandemic measures producing or reinforcing inequalities—which were not sufficiently considered by the government. Our interviewees did not perceive “the economy” (anymore) in microeconomic terms focussing on firms and businesses that needed saving—and as dichotomic to health. Rather, they started to reflect on the socioeconomic dimensions experienced by themselves or observed in others, such as the risk of poverty due to economic consequential effects of preventive measures.

For state–citizen relations, this is an interesting development. Our findings suggest a form of deliberation and meaningful reflection about notions of health, wellbeing, the economy, fellow citizens and government measures. The governmental framing of health as a biomedical matter that can be quantified in terms of available ICU beds appears unsatisfactory, as psychosocial care as well as social and economic cleavages were subordinate to pandemic control in a narrow sense. This was not in line with newly emerging citizens’ understandings of *biosocial* pandemic citizenship.

Moving forward, a critical conceptualisation of health as the focus point of all policies might contribute to the development of policies that reflect the varied everyday practices and experiences of citizens as they navigate the pandemic crisis. The unequal distribution of burdens, challenges and capabilities to comply with pandemic policies during the health emergency marks what we call a social pandemic. Our interviewees pointed to the urgency of taking into account peoples’ diverging positionalities and the challenges they face during the pandemic. They highlight the multidimensional dimensions of health encompassing biopsychosocial and socioeconomic aspects and formulate a notion of *biosocial* citizenship in times of crisis.

## Appendix

See Table 1.

**Table 1:** Demographic characteristics of interviewees

	T1	T2
Total number of interviewees (*n*)	80	72
Age		
18–30	14 (18%)	13 (18%)
31–45	16 (20%)	15 (21%)
46–60	24 (30%)	22 (31%)
61–70	20 (25%)	17 (24%)
70 +	6 (8%)	5 (7%)
Gender		
Female	44 (55%)	41 (57%)
Male	36 (45%)	31 (43%)
Other	0 (0%)	0 (0%)
Household		
Single	20 (25%)	19 (26%)
Couple	35 (44%)	31 (43%)
Living with child(ren) < 12	8 (10%)	7 (10%)
Living with child(ren) 12 +	11 (14%)	10 (14%)
Other	6 (8%)	5 (7%)
Rural/urban		
Big town (e.g. capital, + 500k)	43 (54%)	38 (53%)
Medium/small town	19 (24%)	17 (24%)
Rural (e.g. village)	18 (23%)	17 (24%)
Employment status		
Employed (long-term contract)	30 (38%)	27 (38%)
Self-employed	15 (19%)	14 (19%)
Employed (short-term/precarious contract)	5 (6%)	3 (4%)
Unemployed	5 (6%)	5 (7%)
Retired	19 (24%)	17 (24%)
Other	6 (8%)	6 (8%)
Education		
Less than 10 years	8 (10%)	7 (10%)
10–14 years (e.g. highschool diploma)	27 (34%)	23 (32%)
Higher education	45 (56%)	42 (58%)
Household net income		
Up to 1400€ per month	9 (11%)	9 (13%)
1401–3000€ per month	29 (36%)	27 (38%)
More than 3000€ per month	42 (53%)	36 (50%)

## Footnotes

1 In May 2019, a secretly recorded video was released that was followed by the resignation of the Vice Chancellor Strache (right-wing populist Freedom Party (FPÖ) and early elections. The video showed Strache and an FPÖ deputy leader in a villa in Ibiza, discussing corruption practices and manipulation of mass media in order to influence election results in Austria.

2 Given the specific effects on women—in terms of increases of unpaid work and emotional labour in various contexts of life (e.g. Berghammer 2022)—it is important to note that these roles were all held by men—leading to at times bizarre and war-like settings at press conferences consisting of men in suits ‘commanding’ (as it was perceived by many of our respondents) to population to behave in certain ways.

3 Several ministers stepped back, while criminal charges were prepared against chancellor Kurz who resigned in October 2021. Following a further reshuffling of government officials, the former Minister of Interior Karl Nehammer (ÖVP) became Austria’s new chancellor in December 2021.

4 Trust is measured here on a Likert scale of 0–10 where 0 means “no trust at all” and 10 means “a lot of trust”. The cited numbers of 75% and 30% refer to the numbers of respondents who chose values of 6 to 10 on that scale.

5 The pseudonyms for the interviews were devised as follows: the first letter and number indicate the interview phase in which the interview was conducted (T1 = interview phase 1; T2 = interview phase 2); these are followed by the initials of the person who conducted the interview and a number indicating the progressive order of the interview (e.g. 05 is the fifth interview that XX conducted). For peer review purposes the initials of the person who conducted the interview have been omitted in the final version of the manuscript. In Consequence, 'XX' can stand for various interviewers and for instance 'XX 08' isn’t necessarily the same interview.

6 In April 2020, 71.6% of study respondents to the Austrian Corona Panel Project (ACCP) deemed government measures to contain the spread of COVID-19 “appropriate”. This number decreased to 36.8% in October 2021 (Kalleitner and Partheymüller 2020; Kalleitner et al. 2021).

7 This formulation is inspired by the Department of Global Health and Social Medicine at King’s College London, whose motto has long been that “health is more than a medical matter”. See https://www.kcl.ac.uk/ghsm.
